# Series-Biased Micro-LED Array for Lighting, Detection, and Optical Communication

**DOI:** 10.3390/nano14030307

**Published:** 2024-02-03

**Authors:** Qian Fang, Xiaoxiao Feng, Huiping Yin, Zheng Shi, Feifei Qin, Yongjin Wang, Xin Li

**Affiliations:** GaN Optoelectronic Integration International Cooperation Joint Laboratory of Jiangsu Province, Nanjing University of Posts and Telecommunications, Nanjing 210003, China; 1222014633@njupt.edu.cn (Q.F.); 1222014634@njupt.edu.cn (X.F.); 1223014316@njupt.edu.cn (H.Y.); shizheng@njupt.edu.cn (Z.S.); qinfeifei@njupt.edu.cn (F.Q.); wangyj@njupt.edu.cn (Y.W.)

**Keywords:** multiple quantum wells, III-nitride, micro-LED, visible light communication

## Abstract

Micro-LED arrays exhibit high brightness, a long lifespan, low power consumption, and a fast response speed. In this paper, we have proposed a series-biased micro-LED array by using a nitride layer with multi-quantum wells epitaxial on sapphire substrate. The III-nitride multiple quantum wells serving as the micro-LED active material enable both luminescence and detection functionalities. The micro-LED array combines lighting, detection, and communication capabilities. We have conducted a thorough analysis of the micro-LED array’s optoelectronic features in both lighting and detection modes. We also explore visible light communication performance across different arrangements of single micro-LED devices within the series-biased array. Our research achieves 720p video transmission via visible light communication using the micro-LED array, supporting a communication rate of up to 10 Mbps. Our contributions encompass the successful integration of lighting and detection functions and a comprehensive assessment of optoelectronic and communication performance. This study highlights the multifunctional micro-LED array’s potential as a transceiver terminal in visible light communication systems, expanding its applications from smart lighting to visible light communication and photonic integrated chips. These innovations enhance our understanding of micro-LED technology and its versatile applications.

## 1. Introduction

Owing to its low power consumption, fast response time, and remarkable luminous efficiency, micro-LED arrays based on gallium nitride (GaN) have emerged as a focal point of interest among researchers, particularly in the fields of high-speed visible light communication and cutting-edge lighting technologies [1,2,3,4,5]. These micro-LED arrays, each incorporating numerous micro-LEDs on a single chip, have the potential to improve optical communication. The enhanced communication rates of micro-LED arrays are attributed to their lower RC constants, which are crucial for high-speed data transmission in applications like free-space communication [6,7]. Additionally, the higher operating current densities of these arrays significantly increase the overall light emission intensity, thereby extending communication distances in underwater environments, making them particularly beneficial for underwater communication [8]. The multifunctional capabilities of micro-LEDs position them as promising components in the emerging landscape of IoT systems and smart cities.

The III-nitride material, representing a significant advancement in semiconductor materials as a third-generation compound, offers a highly promising future. Optoelectronic devices based on III-nitride have found widespread application in the fields of illumination, imaging, and optical communication. This prominence is largely attributed to the remarkable optoelectronic characteristics, high breakdown strength, and superior thermal conductivity that III-nitride materials exhibit [9,10,11]. Within the visible spectrum, III-nitride has firmly established itself as the material of choice for LEDs, owing to its stability of light output and extensive spectral coverage [12,13]. Furthermore, III-nitride LEDs offer distinct advantages, including low energy consumption, extended operational lifetimes, and eco-friendly attributes [14].

With the increasing demand for IoT technologies that necessitate compact and energy-efficient terminals, there has been a rapid development in photonics-integrated system-on-a-chip. This technology combines various photonic components, such as light-emitting diodes (LEDs), photodiodes (PDs), and waveguides, on a single photonics chip [15,16]. The development of multifunctional micro-LED arrays capable of both emitting and detecting visible light has the potential to enhance the integration level of photonics chips. Such integrated photonics chips can be used in diverse applications, ranging from smart vehicles to smart cities and healthcare technologies [17]. While micro-LED technology has predominantly found applications in display, in recent years, we have witnessed a surge in research aimed at enhancing critical parameters for micro-LED displays. These improvements encompass augmenting the display size, elevating yield rates, and extending the color gamut coverage [18,19,20]. The achievement of these objectives often hinges on the deployment of mass transfer techniques, which are crucial for enhancing both the display size and yield rates.

However, despite the aforementioned advancements, there is still considerable scope for enhancing micro-LED array research. Micro-LED array chips typically require various components to fulfill distinct functions like illumination and detection. Several methods have been explored for the mass assembly of micro-LEDs, including techniques such as elastomer stamping [21], electrostatic transfer [22], laser-assisted transfer [23], and fluid self-assembly [24]. In a noteworthy development, researchers have successfully realized full-color micro-LED arrays by merging III-nitride semiconductors, perovskite, and quantum dot materials, complemented with advanced color-conversion techniques [25,26]. Moreover, the application of micro-LED arrays as transmitters for visible light communication has emerged as a significant focus. Currently, micro-LED transmitters utilizing MIMO (multiple-input, multiple-output) technology have demonstrated the potential to achieve Gbps-speed visible light communication [27,28,29].

The coexistence of visible light emission and detection capabilities in III-nitride multiple quantum wells materials makes them a prime candidate for integrated photonics chips with diverse recombination functions, exhibiting outstanding performance in lighting, detection, and optical communication [30]. There exists an overlap region between the emission spectrum and the detection spectrum of III-nitride multiple quantum wells materials in the visible range [31].

Utilizing the distinctive characteristics of III-nitride multiple quantum wells, our research presents a series-biased micro-LED array epitaxially grown on a sapphire substrate. This micro-LED array seamlessly integrates lighting, detection, and optical communication functions, offering a versatile platform with multifunctionality. We comprehensively assess the micro-LED array’s optoelectronic performance in both lighting and detection modes, highlighting its multifunctional potential. Our study extensively explores the visible light communication performance across various configurations of single micro-LED devices within the series-biased array. More specifically, we achieve 720p video transmission through visible light communication by using this micro-LED array. The micro-LED array’s role as a transceiver terminal within visible light communication systems further broadens its application horizons. This study, grounded in micro-LED technology, represents a significant stride towards enhanced integration, multifunctionality, and efficiency within the realms of optoelectronics and photonics chips.

## 2. Design and Fabrication of Micro-LED Array

### 2.1. Micro-LED Array Design and Fabrication Process

**Figure 1:**
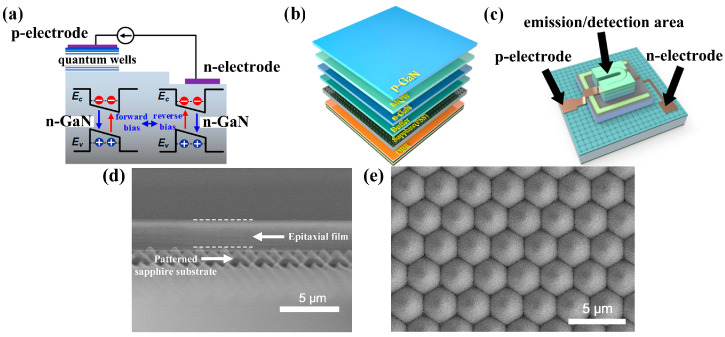
(**a**) Schematic of luminescence detection coexistence of III-nitride multi-quantum wells diode device; (**b**) layered structure of III-nitride epitaxial material with multi-quantum wells on sapphire substrate; (**c**) schematic of single micro-LED device; (**d**) cross-sectional SEM image of the epitaxial film on the patterned sapphire substrate; (**e**) top-view SEM image of the patterned sapphire substrate.

As illustrated in Figure 1a, the diode device, based on III-nitride multi-quantum wells, accomplishes the emission of visible light signals when applied to forward bias. In contrast, when a reverse bias is applied to a diode device with the same structure, the nitride multi-quantum wells transition into a detection mode. In this mode, they absorb photons, leading to the generation of electron–hole pairs and the subsequent production of photocurrents. The seamless transition between electro-optic and optoelectronic bidirectional energy and information exchange is achieved within the unified framework of III-nitride multi-quantum wells integration.

The layered structure of the III-nitride epitaxial films with multi-quantum wells on the sapphire substrate (Xiamen Changelight Co., Ltd., Xiamen, China) is shown in Figure 1b, from top to bottom, as follows: (1) The epitaxial films are grown using metal–organic chemical vapor deposition (MOCVD) on a 4-inch sapphire substrate. The epitaxial films consist of 1.4 μm n-GaN, 500 nm InGaN/GaN MQWs, and 350 nm p-GaN. (2) The substrate layer is sapphire (patterned sapphire substrate, PSS). The patterned substrate could reduce the material defects caused by the different lattice constants and thermal expansion coefficients between the sapphire substrate and the III-nitride epitaxial layer. A cross-sectional SEM image of the epitaxial film on the patterned sapphire substrate is shown in Figure 1d, and a top-view SEM image of the patterned sapphire substrate is shown in Figure 1e. (3) As for the Bragg reflector layer (DBR), made of silicon dioxide (SiO_2_) and titanium dioxide (TiO_2_) alternating layers periodically, its thickness and period are determined by the emission wavelength. The thickness of each layer is 1/4 of the emission wavelength. The DBR is used as a reflector, with high reflectivity for emitting light, and it reduces the light leakage and improves the optoelectronic performance of micro-LEDs. The DBR reflector was pre-evaporated on the bottom of the materials. The DBR (distributed Bragg reflection) layers with 2-μm thickness consist of 13 pairs of periodically arranged SiO_2_ and TiO_2_ layers. In order to obtain high reflectivity for the target wavelength, the DBR has an inhomogeneous thickness distribution of the SiO_2_/TiO_2_ pairs. The thickness of the SiO_2_ layer is 40–90 nm, and the thickness of the TiO_2_ layer is 30–120 nm.

Figure 1c illustrates the structure of a single micro-LED, with the active region being composed of III-nitride multiple quantum wells, enabling dual functionality in emitting and detecting visible light. The application of bias voltage to the active region is achieved through the positive and negative electrodes. With the positive bias, the micro-LED serves as a source of visible light, emitting signals as a lighting unit. Conversely, under the negative bias, the micro-LED operates as a receiver of visible light signals, effectively converting them into a photocurrent as a detection unit.

**Figure 2:**
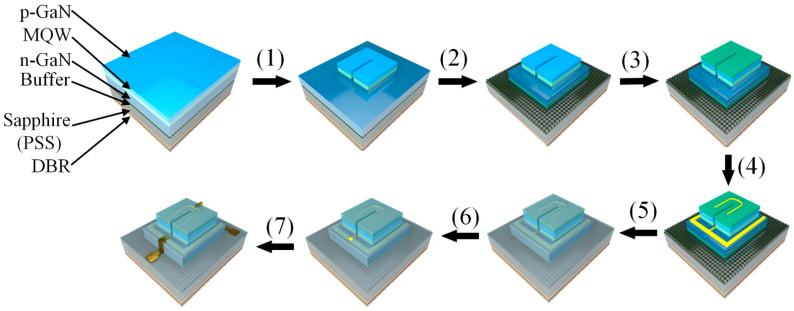
Fabrication process of micro-LED array.

As shown in Figure 2, the fabrication process of the micro-LED array using standard semiconductor processes is as follows: (1) Pattern the structure of the active region on a positive photoresist layer (AZ5214) by photolithography and transfer the structures to the III-nitride epitaxial layer by inductively coupled plasma etching (ICP). (2) Pattern the structure of the whole micro-LED on a positive photoresist (AZ4620) and transfer the structures to the III-nitride epitaxial layer by ICP etching. The etching step penetrates the II-nitride layer to realize the electrical isolation of different micro-LEDs. (3) The 230-nm-thick ITO current spreading layer is prepared by magnetron sputtering and wet etching with the HCl and FeCl_3_ solution. (4) The Ni/Al/Ti/Pt/Au positive and negative metal electrodes are prepared by the lift-off process. The lift-off process is achieved by using the reverse photoresist technique with the AZ5214 photoresist, where the resistance is patterned, followed by Ni/Al/Ti/Pt/Au deposition, and then the resistance is lifted off to leave behind the desired pattern of positive and negative metal electrodes. (5) Employ plasma-enhanced chemical vapor deposition (PECVD) to deposit a 1.5-μm-thick SiO_2_ layer, followed by spin-coating a positive photoresist (AZ4620). After patterning the photoresist, RIE etching is used to pattern SiO_2_, achieving planarization after the ICP etching process. (6) and (7) The pad electrode Ni/Al/Ti/Pt/Au is prepared by the lift-off process. The lift-off process is achieved using the reverse photoresist technique with AZ5214 photoresist, where the resistance is patterned, followed by Ni/Al/Ti/Pt/Au deposition, and then the resistance is lifted off to leave behind the desired pattern of pad electrodes.

### 2.2. Micro-LED Array Morphology Characterization

**Figure 3:**
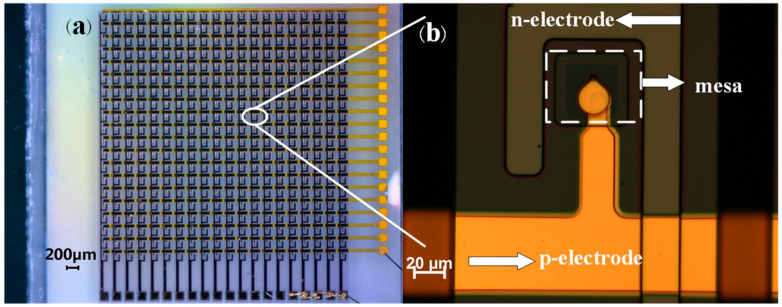
(**a**) Optical microscopy image of 20 × 20 series-biased micro-LED array; (**b**) magnified optical microscope image of a single micro-LED.

Figure 3 displays optical microscopy images of the 20 × 20 series-biased micro-LED array. In Figure 3a, an overall optical microscopy view of the micro-LED array is presented. This array comprises 20 rows and 20 columns of individual micro-LEDs. The positive and negative electrodes of the micro-LEDs are, respectively, connected to the array’s side, forming various series connections of micro-LEDs in terms of their numbers. Figure 3b shows a magnified optical microscope view of a single micro-LED. The active region (emission area) of each micro-LED is a 50 µm × 50 µm square structure. The micro-LEDs are arranged with a pitch of 200 µm. These dimensions were set to optimize the optical performance of the array and to minimize potential optical crosstalk, which is particularly important for applications in visible light communication. This substantial pitch ensures sufficient spatial separation between the adjacent LEDs, effectively minimizing the possibility of optical crosstalk. The positive and negative electrodes, along with the active region LED (mesa structure), are clearly visible and exhibit no apparent defects. These observations signify that the micro-LED array is fabricated with high-quality standards.

## 3. Optoelectronic Properties of Series-Biased Micro-LED Array

**Figure 4:**
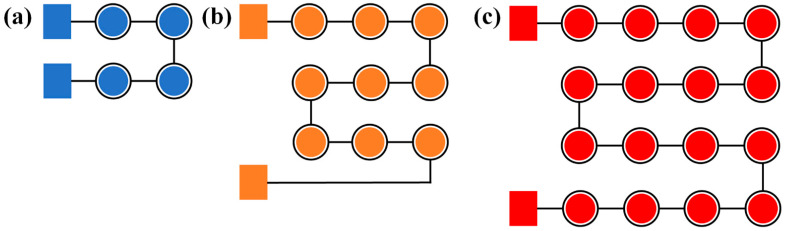
(**a**) Series-biased connected 2 × 2 micro-LED array; (**b**) Series-biased connected 3 × 3 micro-LED array; (**c**) Series-biased connected 4 × 4 micro-LED array.

With an increased number of micro-LEDs connected in a series, the light output power is enhanced; however, this arrangement may lead to a decrease in the rate of visible light communication. Therefore, we have designed the micro-LED array, which is composed of various numbers and forms of single micro-LEDs, to be arranged in a series bias in order to assess their optoelectronic characteristics and visible light communication performance. The purpose of this design is to investigate an optimal balance between the higher light output from more LEDs in the series and the potential reduction in communication rate that such an arrangement could cause. These series connections were realized by wire bonding, using gold wires to directly connect the positive and negative electrodes of the micro-LEDs. Specifically, Figure 4a illustrates a series of four micro-LEDs, Figure 4b shows nine micro-LEDs in a series, and Figure 4c depicts a series of sixteen micro-LEDs. Figure 4a–c are three independent series-connected micro-LED arrays, which are spatially non-overlapping and located in different areas of the layout. Each array is electrically independent in its connecting arrangements.

### 3.1. Electrical Characteristics

**Figure 5:**
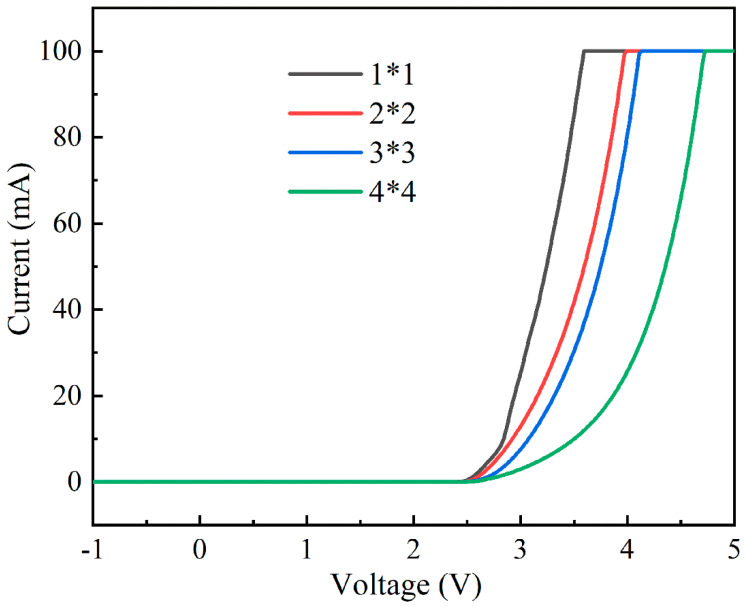
Current–voltage (I–V) characteristics of micro-LED arrays with a different number of series connections.

The electrical characteristics of the series-biased micro-LED array were assessed using an Agilent B1500A (Agilent, Santa Clara, CA, USA) semiconductor device analyzer. These devices operated in lighting mode under positive bias and in detection mode under negative bias, with a voltage range spanning from −5 V to 5 V. There is a safety restriction of the Agilent B1500A that limits the saturation current to 100 mA. Figure 5 illustrates the current–voltage (I–V) curves of the series-biased micro-LED arrays under various series connection arrangements. All of the arrangements exhibited a turn-on voltage of approximately 2.3 V. Notably, minimal current was observed within the negative bias range, demonstrating negligible current leakage in the detection mode. When the voltage reached 3.5 V, the current values of the micro-LED arrays with varying series connection arrangements were as follows: 85 mA (1 × 1), 41 mA (2 × 2), 30 mA (3 × 3), and 10 mA (4 × 4), with ratios of approximately 8.5:4.1:3:1. Ideally, when the same voltage is applied to series-connected LED devices of different quantities, the current ratios should follow a pattern of (1 × 1):(2 × 2):(3 × 3):(4 × 4) = 16:4:1.7:1. This difference between the actual current ratios and the ideal current ratios may be due to factors such as the resistance of the LED contacts, the resistance of the internal metal wiring, and the impedance of the test equipment, which can contribute to these deviations.

### 3.2. Optical Characteristics

**Figure 6:**
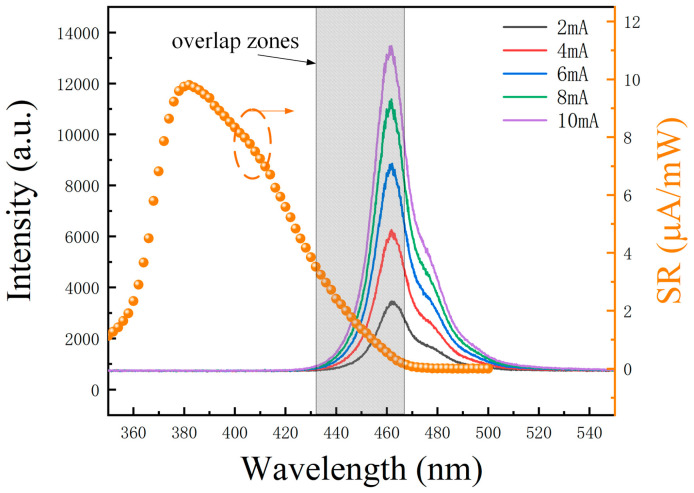
Electroluminescence spectra and responsivity spectra of micro-LED at different currents.

Figure 6 shows the electroluminescence and responsivity spectra of the micro-LED at varying currents. The 4 × 4 series-biased micro-LED was driven by the Keithley 2636B digital source meter, ranging from 2 mA to 10 mA. The emitted light was collected with an optical fiber and directed to the spectrometer. The electroluminescence spectra are depicted by five Gaussian curves in different colors, illustrating the micro-LED’s performance in lighting mode. The peak wavelength fell within the blue range at 461.71 nm, with a full width at half maximum (FWHM) of 17.64 nm. The light intensity exhibited a linear modulation response to the applied currents, rendering the micro-LED an excellent candidate as a transmitter terminal in visible light communication systems. On the right axis of Figure 6, the orange dotted curve represents the responsivity spectra of the 4 × 4 series-biased micro-LED when operating in detection mode. These measurements were obtained using the IQE-200B quantum efficiency measurement system. The peak wavelength for the responsivity spectra was 381.98 nm, boasting a maximum responsivity of 9.80 μA/mW. The responsivity spectra showed a cutoff wavelength of 468 nm. The shaded area in Figure 6 represents an overlap region of approximately 25 nm, where the electroluminescence spectra align with the responsivity spectra. This overlap indicates the electroluminescence spectra in lighting mode and the responsivity spectra in detection mode. Within this spectral overlap, the micro-LEDs can function as transceivers.

The micro-LED devices employed in this study share an identical MQWs structure for both lighting and detection. Within the spectral overlap, the emitted light is converted into photocurrent within the MQWs of the micro-LED in detection mode. Nevertheless, this concurrent emission and detection approach presents limitations in terms of speed and responsivity. Currently, micro-LED receivers cannot match the performance of photodiodes that are specifically tailored for visible light detection. However, in applications such as visible light communication and sensing that necessitate visible light detection, the use of dedicated photodiodes becomes unnecessary. This transition reduces the optical system cost, size, and power consumption, while enhancing the overall system integration in terms of the integrated photonics chip. Our forthcoming research endeavors will explore techniques like selective growth, quantum dot materials, and other approaches to modify the MQWs properties of the micro-LED receiver. These efforts aim to broaden the spectral overlap range, thereby improving its speed and responsivity.

**Figure 7:**
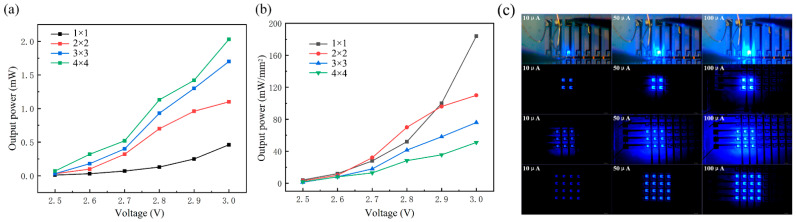
(**a**) Light output power and (**b**) light output power density of micro-LED arrays with a different number of series connections; (**c**) optical microscopy images of micro-LED arrays with a different number of series connections working in lighting mode.

The light output power of the micro-LED array was measured within a bias range of 2.5 V to 3.0 V using a Thorlabs PM100A analog pointer laser power meter, as shown in Figure 7a. The optical power meter probe was fixed at a 3-mm distance perpendicular to the emitting area of the micro-LED array. With increasing voltage, the light output power of the devices connected in different series arrangements exhibited nearly linear growth. This observation emphasizes the strong electro-optic conversion performance of the varying series-connected device numbers. The more micro-LEDs connected in series, the higher the light output power. Nevertheless, under the same driving bias, a larger number of micro-LEDs connected in series leads to increased resistance, resulting in a reduced driving current. Consequently, as shown in Figure 7b, as the number of micro-LEDs connected in series rises, the output power density at the same bias experiences a certain degree of decrease. Figure 7c presents optical microscopy images of the micro-LED array with different series connections operating in lighting mode within a dark environment, illuminated by injection currents ranging from 10 μA to 100 μA. With an increase in current, the light output power from various series connections experiences a substantial rise. Notably, no defective pixels are observed in Figure 7c. The micro-LEDs operating in lighting mode exhibit clarity and brightness, devoid of inter-device interference.

## 4. Visible Light Communication Characteristics of Series-Biased Micro-LED Array

### 4.1. Visible Light Communication and Video Transmission as Transmitter

**Figure 8:**
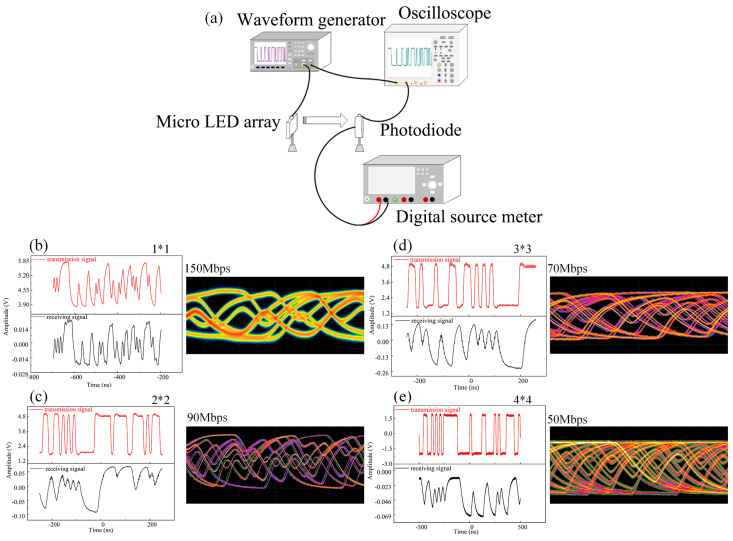
(**a**) Visible light communication test system with a series-biased micro-LED array as a transmitter. Waveform and eye diagrams of micro-LED arrays with 1 × 1 (**b**), 2 × 2 (**c**), 3 × 3 (**d**), and 4 × 4 (**e**) series numbers.

Figure 8a presents the schematic of an experimental setup for visible light communication using a series-biased micro-LED array as the transmitter. To characterize the digital signal transmission capabilities, a pseudo-random bit sequence (PRBS) generated with an Agilent 33522A signal generator is utilized. This sequence, known for its effectiveness in simulating a wide range of data patterns, is applied to the micro-LED array. The modulated optical signal is then captured at an 80-cm distance with a highly sensitive avalanche photodiode (Hamamatsu, C12702-12). The entire setup is linked to an Agilent DSO9254A digital storage oscilloscope, which facilitates a detailed analysis by comparing the original transmitted signal to the received one.

Subsequently, Figure 8b–e show the waveform and eye diagrams corresponding to the micro-LED arrays with an increasing number of series connections. The red lines in Figure 8 represent the transmission signal, while the black lines represent the receiving signal. The waveform and eye diagrams become progressively distorted as the number of series connections increases. The increase in resistance and capacitance with the larger number of series connections leads to a slower response time and reduced bandwidth, causing the signal’s shape to deteriorate, particularly at higher data rates, which is evident in the observed distortion of the waveform and degradation of the eye diagram clarity. Additionally, the increased thermal effect resulting from the series connection of multiple units may also contribute to this phenomenon. The data in Figure 8 illustrate the operational thresholds of the micro-LED array and the upper bounds of data transmission capabilities achievable before significant performance deterioration. The upper bounds vary with the number of series connections, as follows: a 1 × 1 array achieves 150 Mbps, a 2 × 2 array manages 90 Mbps, a 3 × 3 array sustains 70 Mbps, and a 4 × 4 array is capable of transmitting at 50 Mbps. Even at 50 Mbps, the 4 × 4 array sustains functionality suitable for practical applications in intelligent lighting and visible light communication systems, such as those used in audio and video transmission.

**Figure 9:**
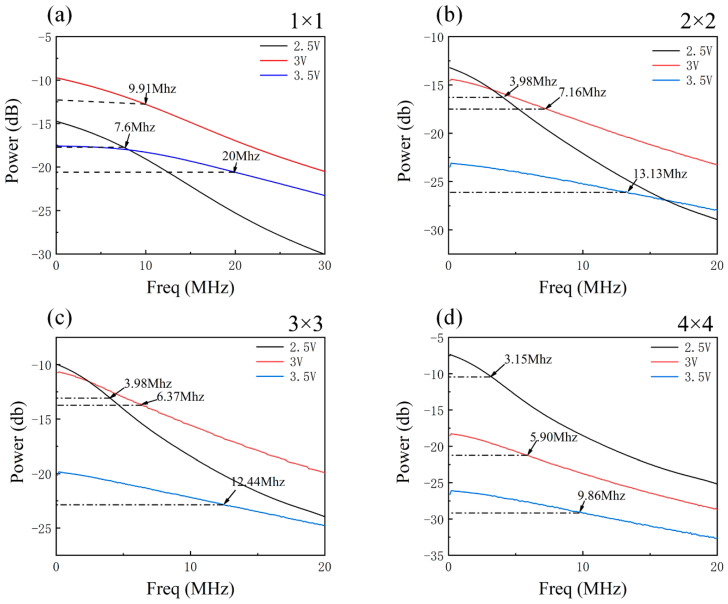
Micro-LED arrays, of 3 dB bandwidth as the applied voltage varies from 2.5 V to 3.5 V, with 1 × 1 (**a**), 2 × 2 (**b**), 3 × 3 (**c**), and 4 × 4 (**d**) series numbers.

Figure 9 displays the 3 dB bandwidth of the micro-LED array with varying numbers of series connections, measured using the vector network analyzer Keysight E5080A. The 3 dB bandwidth exhibits a gradual increase as the applied voltage varies from 2.5 V to 3.5 V. This phenomenon can be attributed to the growing carrier density within the quantum well, which, in turn, shortens the carrier lifetime and elevates the communication response rate. Conversely, as the number of series connections increases, there is a nonlinear rise in parasitic resistance and capacitance. Consequently, the RC constant increases, causing a reduction in the 3 dB bandwidth. This behavior indicates that the communication response performance of the micro-LED array improves with a decrease in the number of series connections. For example, at a voltage of 3.5 V, the 3 dB bandwidth varies for devices from 1 × 1 to 4 × 4, measuring 20 MHz, 13.13 MHz, 12.44 MHz, and 9.86 MHz, respectively.

**Figure 10:**
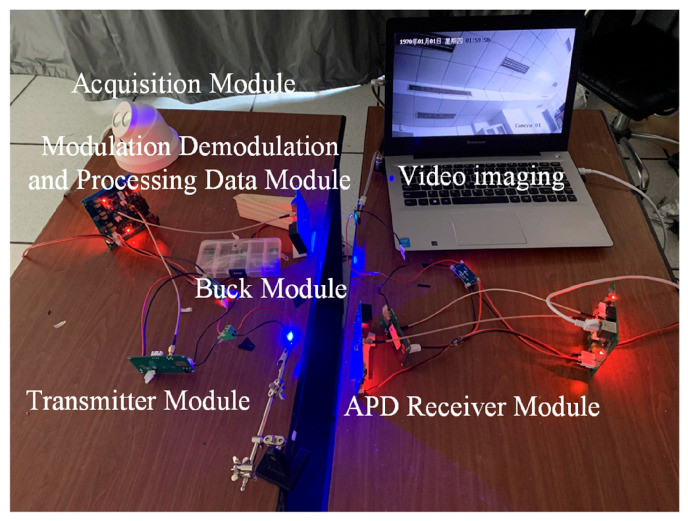
Video transmission system of visible light communication.

In Figure 10, we illustrate a visible light communication (VLC) system specifically designed for video transmission. This system integrates a camera that captures video data, which are then transmitted via a micro-LED array serving as the VLC transmitter. The transmitted visible light signal carries the video data to an avalanche photodetector (APD), which acts as the receiver, converting the light signal back into an electrical form. The modulated data are then restored through post-amplification and shaping circuits, demodulated, and sent to a host computer via Ethernet for real-time display. This setup successfully transmitted a high-definition 720p video at a bitrate of 10 Mbps through the VLC system, demonstrating clear and smooth video playback with minimal latency. An interesting aspect of this system is its response to interruptions in the light transmission path. For instance, when the VLC path is obstructed, the video display on the host computer halts, and promptly resumes normal playback once the obstruction is removed. This observation underlines the micro-LED array’s effectiveness in real-time video transmission and its potential application in intelligent lighting systems. The experiment illustrates the potential of the micro-LED array in real-world applications for visible light communication systems, such as those used in audio or video transmission, going beyond mere data transmission rates or frequencies. Please refer to the Supplementary Materials for an experimental video, which serves as a practical demonstration of the micro-LED array’s capabilities in video transmission.

### 4.2. Visible Light Communication Testing as Transceiver

**Figure 11:**
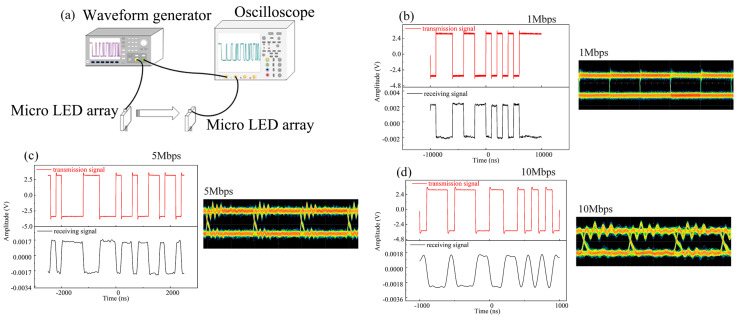
(**a**) Visible light communication test system with series-biased micro-LED arrays as transceivers;(the waveform and eye diagram of a micro-LED array with a 4 × 4 series as a transmitter/micro-LED array with a 2 × 2 series as a receiver with transmission rates of 1 Mbps (**b**), 5 Mbps (**c**), and 10 Mbps (**d**).

The visible light communication characteristics of the series-biased micro-LED array, acting as a transceiver, were investigated using the system illustrated in Figure 11a. Employing a pseudo-random bit sequence generated by the signal generator, the micro-LED array with a series arrangement of 4 × 4 served as the transmitter. The modulated light was then collected by the micro-LED array with a series arrangement of 2 × 2, acting as the receiver, situated at an 80-cm distance in free space. Both the generator signal and the electrical signal converted by the receiver were connected to the Agilent DSO9254A digital storage oscilloscope for a comparative analysis of the transmitted and received signals in visible light communication. Figure 11b depicts the waveform and eye diagram corresponding to transmission rates of 1 Mbps, 5 Mbps, and 10 Mbps, respectively. The visible light communication performance of the micro-LED array as a transceiver is robust and consistent. As the transmission rate increased to 10 Mbps, the waveform exhibited degradation, and the eye diagram became distorted. This indicates that the transmission rate of the micro-LED array, functioning as an integrated transceiver, is capable of reaching up to 10 Mbps. Beyond its conventional applications in lighting, the micro-LED array can fulfill the role of a transceiver in the visible light communication system, facilitating the transmission of audio and video data, environmental sensing data, positioning data, and more.

## 5. Conclusions

In conclusion, this paper introduces and realizes a series-biased micro-LED array that seamlessly integrates lighting, detection, and communication functions within a single chip. This integration is made possible by leveraging the co-existence of the emission and detection properties inherent in III-nitride multi-quantum wells. We have comprehensively characterized the optoelectronic and communication performance of the integrated micro-LED arrays across various biasing conditions and series interconnection arrangements. A significant finding is the 25-nm spectral overlap observed between the electroluminescence spectrum of the micro-LED operating as a transmitter and the responsivity spectra of the micro-LED operating as a receiver. The waveform and eye diagrams become progressively distorted as the number of series connections increases. The upper bounds vary with the number of series connections, as follows: a 1 × 1 array achieves 150 Mbps, a 2 × 2 array manages 90 Mbps, a 3 × 3 array sustains 70 Mbps, and a 4 × 4 array is capable of transmitting at 50 Mbps. The 3 dB bandwidth, assessed under different bias voltages and series connection numbers, ranged from 9.86 to 20 MHz. A visible light communication link with a speed of 10 Mbps and the transmission of high-quality 720p video with a 10-Mbps bitrate were achieved by using a 4 × 4 transmitter and 2 × 2 receiver arrangements, demonstrating the practical utility of micro-LED arrays.

With their compact and highly integrative capabilities, series-biased micro-LED arrays emerge as promising candidates for enabling miniature smart photonic systems and networks for future advancements in lighting and visible light communication. Our ongoing research will prioritize the refinement of micro-LED arrays for specific applications, with a focus on enhancing the spectral overlap between emission and detection for an improved overall performance.

## Data Availability

Archived data sets can be found at GaN Optoelectronic Integration International Cooperation Joint Laboratory of Jiangsu Province, Nanjing University of Posts and Telecommunications.

## Supplementary Materials

The following supporting information can be downloaded at: https://www.mdpi.com/article/10.3390/nano14030307/s1; Video S1: Video transmission of visible light communication.
